# Health literacy among long-term survivors of breast cancer; exploring associated factors in a nationwide sample

**DOI:** 10.1007/s00520-022-07183-3

**Published:** 2022-06-08

**Authors:** Kathrine F. Vandraas, Kristin V. Reinertsen, Cecilie E. Kiserud, Synne K. Bøhn, Hanne C. Lie

**Affiliations:** 1 National Advisory Unit for Late Effects After Cancer Treatment, Department of Oncology, Oslo University Hospital, Radium Hospital, Oslo, Norway; 2 Department of Behavioral Sciences in Medicine, Institute of Basic Medical Sciences, Faculty of Medicine, University of Oslo, Oslo, Norway

**Keywords:** Health literacy, Breast cancer survivorship, Neuroticism, Fear of cancer recurrence

## Abstract

**Background:**

Poor health literacy may hamper health management and long-term outcomes in breast cancer survivorship. Knowledge of factors associated with poor health literacy is needed to identify survivors in need of additional support and to improve the quality of health care, but is currently scant. Here, we explore health literacy and associated factors in a nationwide sample of long-term survivors of breast cancer.

**Material and methods:**

All survivors aged 20–65 years when diagnosed with stage I–III breast cancer in 2011 or 2012 were identified through the Norwegian Cancer Registry, and invited to participate in the Survivorship, Work and Sexual Health (SWEET) study. Health literacy was measured using The European Health Literacy Survey Questionnaire-12 (HLS-EU-Q12) and analyzed as a continuous and categorical variable. Associations between health literacy and socioeconomic, physical, and mental health variables, including the most common late effects after cancer treatment, were explored in uni- and multivariable linear regression models.

**Results:**

The final sample consisted of 1355 survivors (48%) with a mean age of 60 years at survey (SD 8.7). Eight years had passed since diagnosis (SD.0.7), and the majority of survivors had high socioeconomic status. Advanced judgment calls concerning treatment and health risks were reported to be the most difficult for survivors to handle. Mean health literacy sum score was 36.2 (range 12–48, SD 5.4). Thirty-nine percent had intermediate, while 19.3% reported marginal or inadequate health literacy. Education, income, age at diagnosis, the personality trait neuroticism, and fear of cancer recurrence were significantly associated with health literacy in the multivariate model, explaining 12% of the variance in health literacy scores.

**Conclusion:**

Low levels of health literacy were prevalent in this population-based sample of long-term survivors of breast cancer, despite high socioeconomic status. Communicating and interpreting risks seem to be especially challenging. Attention to health literacy at a societal and individual level is necessary in order to provide survivorship care of high quality.

## Background

Health literacy (HL) is the ability to access, understand, appraise, and apply health information [1], and effect long-term health outcomes [2, 3]. Within various chronic conditions, individuals with low HL are reported to have higher mortality, more frequent use of health care services, and reduced quality of life compared to individuals with high HL [4, 5]. Almost 1 in 2 Europeans is reported to have limited HL, but there are extensive variations in prevalence likely reflecting a social gradient [2, 6]. HL is especially relevant in an oncological setting given the sheer complexity of diagnostics and treatments, and the expectations of active patient involvement [7]. Despite the importance of sound knowledge of HL in oncology, research is limited compared to other major illnesses.[8].

Self-management encompasses individuals’ ability to monitor their health and manage cognitive, behavioral, and emotional responses to preserve quality of life [9], and has been identified as a possible mediator between HL and poor health outcomes [10]. Low HL and inefficient self-management skills are associated with increasing symptom burden [11] highlighting the importance of such skills for cancer survivorship. A recent study on survivors of head and neck cancer reported lower levels of self-management behaviors and health-related quality of life among survivors with low HL compared to individuals with high HL [12].

Patients with early-stage breast cancer have excellent long-term prognosis with 5-year survival rates above 90% in the Western World [13], resulting in an increasing population of survivors of breast cancer. Survivorship is characterized by substantial uncertainty due to risks of cancer recurrence and treatment-related late effects (LEs), such as fatigue, cognitive dysfunction, mental health problems, sleep disturbances, neuropathy, and pain [14, 15]. To mitigate these risks, survivors are recommended to adhere to a healthy lifestyle and follow-up guidelines. As such, cancer survivorship resembles that of other chronic diseases, including type 2 diabetes, where effective self-management is associated with improved health outcomes [4].

Among patients with breast cancer, low HL appears to be prevalent with estimates varying between 50 and 90%, with highest estimates among elderly and in lower socioeconomic groups [16, 17]. Survivors of breast cancer with low HL report more fear of cancer recurrence (FCR) [16], a higher degree of unmet informational and supportive care needs [18], and lower health-related quality of life compared to survivors with high HL [11].

To provide sufficient and adjusted support to individuals with low HL and to increase HL and thereby self-management are potentially potent pathways for improving long-term health outcomes among survivors of breast cancer. In order to succeed, we need to be able to identify individuals with low HL. Such knowledge may also stimulate efforts at a societal and health care level aimed at improving informational provision and communication. The aims of this study were to describe HL in a large, nationwide cohort of long-term survivors of breast cancer and to explore factors associated with HL, including the most prevalent physical and mental LEs.

## Material and methods

### Sampling

All Norwegian female survivors of breast cancer aged 20–65 years when diagnosed with breast cancer stage I–III in 2011–2012 were invited to participate in the Survivorship, Work and Sexual Health (SWEET) study, in total 2803 women. Survivors were identified through the Cancer Registry of Norway (CRN) which is based on mandatory reporting, providing close to complete prevalence estimates [19]. Survivors had to be free of prior or successive malignant disease in order to be included, with the exception of survivors diagnosed with non-melanoma skin cancer or ductal carcinoma in situ in addition to their invasive BC. One reminder was sent to survivors who did not respond to the first invitation (*n* = 1684). The non-responders in the SWEET study were significantly older (53.2 years at diagnosis versus 51.9 years), a larger proportion was lymph node-negative (64% versus 60%) and Her2-negative (80% versus 76%), and Ki67 was lower (mean 27 versus 31). There were no significant differences in tumor size, hormone receptor status, or type of surgery between responders and non-responders (results not shown).

### Primary outcome

*HL* was self-reported using the European Health Literacy Survey Questionnaire-12 (HLS-Q12), a short version of the validated HLS-Q47. The short version consists of 12 items which measure HL across the four cognitive domains (access, understand, appraise, and apply health information) and the three health contexts (health care, disease prevention, and health promotion) capturing the multidimensionality of HL as proposed in the HLS-Q47 [1]. The HLS-Q12 is reported to be a valid screening tool for HL in a clinical setting, and has been validated in the Norwegian population [20]. Answers to each item are given on a 4-point rating scale from very difficult to very easy, resulting in a sum score from 12 to 48, where a higher score reflects higher levels of HL. In addition, a “don’t know” category is included for each item [20]. “Don’t know” responses and missing values were imputed with mean score of all items, if more than 80% of items were completed. In addition to the sum score, HL values were categorized into four levels based on previously defined thresholds: inadequate (12 to 26 points), marginal (27 to 32 points), intermediate (33 to 38 points), and advanced HL (39 to 48 points) [21].

### Variables

#### Self-reported

Socioeconomic variables included education, financial income during the year prior to survey living arrangements, and employment (Table 1). Somatic comorbidity was based on a modified version of the Charlson comorbidity index [22, 23]. The personality trait neuroticism was assessed using a short version of the Eysenck Personality Questionnaire [24]. Missing values were substituted with mean values for that item from the overall sample when more than 50% of items had been answered. Cronbach’s alpha was 0.8.

**Table 1:** Description of study sample (*n*=1355) for categorical (*n* (%)) and continuous (mean, SD and median, range) variables

Categorical variables	*n* (%)	
Socioeconomic variables		
Education ≤ 12 y Education >12 y	647 (47.7) 691 (51.0)	
- ≤4 years higher education - >4 years higher education	290 (21.4) 401 (29.6)	
High household income^a^	923 (68.1)	
Living with a partner or children	1079 (79.6)	
Employment^b^	1035 (76.4)	
Clinical variables		
Receptor expression		
- Hormone receptor positive	1153 (85.1)	
- HER-2 positive	248 (18.3)	
Triple negative	116 (8.6)	
Stage (TNM)^c^		
I II III	606 (44.7) 486 (35.9) 108 (8.0)	
Surgery^d^		
- Mastectomy - Breast-conserving therapy	559 (41.3) 788 (58.2)	
Chemotherapy, all regimens	926 (68.3)	
Radiotherapy, all regimens	1087 (80.2)	
Endocrine therapy, all regimens	881 (65.0)	
Herceptin	242 (17.9)	
Physical health		
Comorbidity		
- 1–2 conditions	731 (53.9)	
- >2 conditions	326 (24.1)	
Neuropathy (SCIN)	277 (20.4)	
Sleep problems (HUNT)^e^	59.2 (43.7)	
Continous variables	**Mean (SD)**	**Median (range)**
Age at survey (years)	59.9 (8.7)	60.0 (30–74)
Age at diagnosis (years)	51.9 (8.6)	52.0 (21–65)
Time since diagnosis (years)	8.0 (0.7)	8.02 (7–9)
Neuroticism (Eysenck Personality questionnaire)^f^	2.17 (1.9)	2.0 (0–6)
Cognitive function (EORTC QLQ-C30)^g^	74.3 (24.9)	83.3 (0–100)
Arm symptoms (EORTC QLQ BR-23)^f^	20.7 (24.6)	11.1 (0–100)
Breast symptoms (EORTC QLQ BR-23)^f^	16.2 (19.3)	8.3 (0–100)
Pain (EORTC QLQ-C30)^f^	28.1 (29.4)	16.7 (0–100)
Depressive symptoms (PHQ-9)^f^	5.9 (4.5)	5.0 (0–27)
Anxiety symptoms (GAD-7)^f^	3.8 (3.6)	3.0 (0–21)
Fear of cancer recurrence (CARQ)^f^	11.7 (8.7)	9.0 (0–40)
Fatigue (Chalder’s fatigue questionnaire)^f^	14.8 (5.8)	13.0 (0–33)

Abbreviations: *HER-2*, human epidermal growth receptor-2; *SCIN*, scale for chemotherapy-induced long-term neurotoxicity; *HUNT*, Nord-Trøndelag Health Study; *EORTC QLQ*, European Organization for Research and Treatment of Cancer quality of life questionnaire; *PHQ-9*, The Patient Health Questionnaire-9; *GAD-7*, General Anxiety Disorder 7-item scale; *CARQ*, Concern About Recurrence Questionnaire. ^a^≥ 500000 NOK/year equal to 48000€/year. ^b^Employment = paid work (full time, part time, on sick leave), self-employed and freelance. ^c^*n*= 1200 due to missing values for 11.4%. ^d^*n*=1347 due to missing values for 0.6%. ^e^Experiencing one or more of the following at least 3 times per week: difficulties falling asleep at night and/or waking up too early without being able to go back to sleep. ^f^Increasing score=increasing symptoms. ^g^Increasing score = increasing function

#### Cancer-related characteristics

Age at diagnosis, pathological stage, hormone receptor and HER-2 status, and information on surgical treatment were obtained from the CRN. Additional treatment information was based on self-report.

#### Possible late effects after breast cancer

Pain intensity and cognitive function were assessed using the European Organization for Research and Treatment of Cancer quality of life questionnaire (EORTC- QLQ - C30 version 3) [25]. Cronbach’s alpha was 0.9. Neuropathy was assessed using the scale for chemotherapy-induced long-term neurotoxicity (SCIN) [26]. Arm and breast symptoms were assessed using the European Organization for Research and Treatment of Cancer Breast Cancer–Specific Quality of Life Questionnaire Module (EORTC QLQ-BR23 questionnaire) [27]. Cronbach’s alpha for the BR23 was 0.8. Fatigue was measured using Chalder’s Fatigue Questionnaire [28]. Cronbach’s alpha was 0.9. Sleep problems were defined using two items from the Nord-Trøndelag Health Study (the HUNT-study) [29]. Depressive symptoms were measured using The Patient Health Questionnaire-9 (PHQ-9) [30]. Cronbach’s alpha was 0.8. Anxiety symptoms were evaluated using the General Anxiety Disorder 7-item scale (GAD-7) [31] Cronbach’s alpha was 0.9. FCR was measured using four items from the Concern About Recurrence Questionnaire (CARQ) [32]. Cronbach’s alpha was 0.7 (Table 1).

### Statistical analyses

Descriptive statistics was performed for the sample in total. For the HL variable, response frequencies across the 12 items of the HLS-Q12, in total and within each response category, were performed, before analyzing HL both as a categorical and continuous variable. Mean scores for HL based on imputed and original values were calculated. Univariate linear regression with HL as the dependent variable was carried out, and all statistically significant associations (*p*-value ≤ 0.1) were incorporated into a multivariate linear model. Survivors with one or more missing values on any of the included variables were excluded from the regression analyses. IBM SPSS statistics version 26 (SPSS, Chicago, IL) was used for all analyses.

## Results

In total, 1361 survivors returned the survey (49% response rate) among whom three were excluded due to incomplete consent, and three due to self-reported breast cancer recurrence. The final sample included 1355 (48%) survivors. Mean age at time of survey was close to 60 years (59.9 years, SD 8.7), and on average, eight years (SD 0.7) had passed since diagnosis. More than half of the sample had higher education (51%), including 30% who had more than 4 years of higher education, and 68% had a high household income. At time of diagnosis, 3/4 of the survivors were employed (76.4%). Most had undergone surgical treatment (99.5%), and close to 68% had received chemotherapy. Almost 80% reported at least one comorbid condition (78%). Sleep problems were present among 44% and 20% reported a high degree of neuropathy (Table 1).

The HL item concerning the ability to judge the advantages and disadvantages of different treatment options had the lowest scores, rated as difficult or very difficult by 41.1%, followed by the item assessing the ability to judge if information on health risk in the media is reliable (35.3%). In contrast, the items on ability to follow written instructions on medications and to understand why health screening was important were reported to be easy or very easy by 91.7% and 93.8% respectively (Table 2). HL scores in the inadequate or marginal range were reported among 19.3%, while 39% had intermediate HL. The mean HL sum scores were 36.4 (SD 5.5) before imputation and 36.2 (SD 5.4) after imputation (Table 2).

**Table 2:** Frequencies of responses categorized from very difficult to very easy for each item of The European Health Literacy Survey Questionnaire-12 (range 12–48), categorized health literacy levels from inadequate to advanced, and health literacy sum scores before and after imputation

	*n*: 1355 (%)	Very difficult (1 point)	Difficult (2 points)	Easy (3 points)	Very easy (4 points)	Don’t know
On a scale from very difficult to very easy, how easy would you say it is to:						
*Find information on treatments of illnesses that concern you?*	1324 (97.7)	21 (1.5)	198 (14.6)	744 (54.9)	214 (15.8)	147 (10.8)
*Understand what to do in a medical emergency?*	1311 (96.8)	25 (1.8)	287 (21.2)	664 (49.0)	200 (14.8)	135 (10.0)
*Judge the advantages and disadvantages of different treatment options?*	1247 (92.0)	53 (3.9)	504 (37.2)	455 (33.6)	99 (7.3)	136 (10.0)
*Follow the instructions on medication?*	1323 (97.6)	3 (0.2)	49 (3.6)	742 (54.8)	500 (36.9)	29 (2.1)
*Find information on how to manage mental health problems like stress or depression?*	1319 (97.3)	26 (1.9)	299 (22.1)	592 (43.7)	162 (12.0)	240 (17.7)
*Understand why you need health screenings?*	1331 (98.2)	2 (0.1)	32 (2.4)	635 (46.9)	636 (46.9)	26 (1.9)
*Judge if the information on health risks in the media is reliable?*	1303 (96.2)	47 (3.5)	431 (31.8)	535 (39.5)	157 (11.6)	133 (9.8)
*Decide how you can protect yourself from illness based on advice from family and friends?*	1308 (96.5)	49 (3.6)	333 (24.6)	522 (38.5)	157 (11.6)	247 (18.2)
*Find information on healthy activities, such as exercise, healthy food and nutrition?*	1322 (97.6)	8 (0.6)	69 (5.1)	748 (55.2)	478 (35.3)	19 (1.4)
*Understand information on food packaging?*	1320 (97.4)	14 (1.0)	221 (16.3)	703 (51.9)	347 (25.6)	35 (2.6)
*Judge which everyday behaviour is related to your health?*	1320 (97.4)	7 (0.5)	135 (10.0)	742 (54.8)	412 (30.4)	24 (1.8)
*Make decisions to improve your health?*	1326 (97.9)	23 (1.7)	324 (23.9)	682 (50.3)	275 (20.3)	22 (1.6)
Health literacy, categorized	***n*** **(%)**					
- Inadequate (12–26 points)	31 (2.3)					
- Marginal (27–32 points)	231 (17.0)					
- Intermediate (33–38 points)	528 (39.0)					
- Advanced (39–48 points)	328 (24.2)					
Total	1118 (82.5)^a^					
	**Mean (SD)**					
Health literacy sum score, prior to imputation (range 12–48)	36.4 (5.5)					
Health literacy sum score, after imputation (range 12–48)	36.2 (5.4)					

^a^Missing values (non-responders and “don’t know” responses): 237 (17.5%)

In the univariate analyses, HL was associated with educational attainment (*B* = 2.4, *p* <0.01), age at survey (*B* = −0.07, *p* <0.01), and income (*B* = 2.2, *p* <0.01). Employment was inversely associated with HL (*B* = −1.2, *p* 0.01), as was neuroticism (*B* = −0.67, *p* <0.01). The only cancer-related variables associated with HL were hormone receptor positivity (*B* = −1.2, *p* 0.01) and age at diagnosis (*B* = −0.07, *p* <0.01). Comorbidity (*B* = −1.48, *p* <0.01) and all late effects (pain: *B* = −0.02, *p*-value >0.01; breast symptoms: *B* = −0.04, *p* <0.01; arm symptoms: *B* = −0.03, *p* <0.01; neuropathy: *B* = −0.87, *p* 0.04; sleep problems: *B* = −0.67, *p* <0.01; fatigue: *B* = −0.11, *p* <0.01; depressive symptoms: *B* = −0.21, *p* <0.01; anxiety symptoms: *B* = −0.32, *p* <0.01; and fear of cancer recurrence: *B* = −0.15, *p* <0.01) were inversely associated with HL, except for cognitive function which was positively associated with HL (*B* = 0.03, *p* <0.01) (Table 3).

**Table 3:** Univariate linear regression analysis with health literacy sum score as dependent variable

	Unstandardized coefficient *B*	Standardized coefficient Beta	Sig.^a^	95% CI^b^ for *B*	
				Lower bound	Upper bound
Education^c^	1.09	0.08	**0.02**	0.22	1.96
>4 y. higher education	2.43	0.21	**0.00**	1.67	3.20
Household income^c^	2.21	0.19	**0.00**	1.47	2.94
Living with partner/children^d^	−0.34	−0.03	0.44	−1.21	0.53
Age at survey	−0.07	−0.11	**0.00**	−0.11	−0.03
Employment^d^	−1.16	−0.09	**0.01**	−1.99	−0.33
Age at diagnosis	−0.07	−0.11	**0.00**	−0.11	−0.03
Hormone receptor positivity^d^	−1.21	−0.08	**0.01**	−2.17	−0.24
TNM stage 2 vs 1	−0.59	−0.05	0.11	−1.32	0.14
TNM stage 3 vs 1	−0.40	−0.02	0.54	−1.65	0.86
TNBC^d^	0.80	0.04	0.20	−0.41	2.02
Surgery^d^	−0.36	−0.03	0.30	−1.05	0.33
Chemotherapy^d^	0.49	0.04	0.20	−0.26	1.24
Radiotherapy^d^	−0.43	−0.03	0.32	−1.28	0.41
Endocrine therapy^d^	−0.22	−0.02	0.56	−0.96	0.52
Herceptin^d^	0.59	0.04	0.20	−0.31	1.49
1–2 comorbid conditions^d^	−1.00	−0.09	**0.02**	−1.86	−0.14
>2 comorbid conditions^d^	−1.48	−0.12	**0.00**	−2.47	−0.48
Pain^e^	−0.02	−0.11	**0.00**	−0.03	−0.01
Cognitiv function^f^	0.03	0.16	**0.00**	0.02	0.05
Breast symptoms^e^	−0.04	−0.13	**0.00**	−0.06	−0.02
Arm symptoms^e^	−0.03	−0.14	**0.00**	−0.04	−0.02
Neuropathy^c^	−0.87	−0.07	**0.04**	−1.69	−0.04
Sleep problems^d^	−0.67	−0.06	0.06	−1.37	0.03
Fatigue^e^	−0.11	−0.12	**0.00**	−0.17	−0.05
Depressive symptoms^e^	−0.21	−0.18	**0.00**	−0.28	−0.13
Anxiety symptoms^e^	−0.32	−0.21	**0.00**	−0.41	−0.22
Fear of cancer recurrence^e^	−0.15	−0.23	**0.00**	−0.18	−0.11
Neuroticism^e^	−0.67	−0.24	**0.00**	−0.85	−0.50

^a^Significance level, ^b^confidence interval, ^c^reference low, ^d^reference not, ^e^higher score implies worse symptoms, ^f^higher score implies better function. **Bold:** Statistically significant (*p*<.05) associated with HL

In multivariate analyses, HL was significantly associated with more than 4 years of higher education (*B* =1.32, *p* <0.01), age at diagnosis (*B* = −0.08, *p* <0.01) and income (*B* = 1.1, *p* 0.01). Other socioeconomic variables were no longer significantly associated with HL. FCR and neuroticism remained inversely associated with HL (*B* = −0.08, *p* <0.01, and *B* = −0.27, *p* 0.03 respectively). All other late effects were non-significant in the adjusted analyses. The included explanatory variables collectively explained 12% of the variance in HL (*R*^2^_adj_ 0.12) (Table 4). Age at survey was not included in the analysis due to high collinearity with age at diagnosis.

**Table 4:** Multivariate linear regression analysis with health literacy sum score as dependent variable

	Unstandardized coefficients	SE^a^	Standardized coefficients Beta	Sig.^b^ *R*^2^	*R*^2^_adj_
	*B*			0.14	0.12
Education^c^	0.55	0.44	0.04	0.21	
>4 y. higher education	1.32	0.41	0.12	**0.00**	
Household income^c^	1.11	0.40	0.10	**0.01**	
Employment^d^	0.17	0.41	0.02	0.68	
Age at diagnosis	−0.08	0.03	−0.13	**0.00**	
Hormone receptor positivity^d^	−0.72	0.47	−0.05	0.13	
1–2 comorbid conditions^d^	−0.16	0.44	−0.02	0.72	
>2 comorbid conditions^d^	0.17	0.56	0.01	0.76	
Pain^e^	0.01	0.01	0.06	0.18	
Cognitiv function^f^	0.02	0.01	0.08	0.08	
Breast symptoms^e^	0.00	0.01	−0.01	0.89	
Arm symptoms^e^	−0.02	0.01	−0.07	0.08	
Neuropathy^c^	0.06	0.44	0.00	0.90	
Sleep problems^d^	0.44	0.37	0.04	0.24	
Fatigue^e^	0.01	0.04	0.02	0.74	
Depressive symptoms^e^	−0.01	0.07	−0.01	0.84	
Anxiety symptoms^e^	−0.12	0.08	−0.08	0.12	
Fear of cancer recurrence^e^	−0.08	0.02	−0.13	**0.00**	
Neuroticism^e^	−0.27	0.13	−0.10	**0.03**	

^a^Standard error, ^b^significance level, ^c^reference low, ^d^reference not, ^e^higher score implies worse symptoms, ^f^higher score implies better function. **Bold:** Statistically significant (*p*<.05) associated with HL

## Discussion

Despite high socioeconomic status, one in five long-term survivors of breast cancer had HL levels in the marginal or inadequate range. If not provided with sufficient support, survivors with HL at this level may struggle to understand and make use of health-related information and engage in effective self-management, which are key aspects of successful cancer survivorship.

Three HL items were labelled as difficult or very difficult by less than 10% of the sample, reflecting a high degree of more basic or functional HL skills present in the sample, such as following written instructions. However, 35–41% reported difficulty with advanced HL tasks, such as complex judgment calls regarding treatment and health risks, which reflect the cognitive domain “appraisal” in the original HL framework [1]. These findings support the theory that HL is as a multi-dimensional concept which may vary across health-related contexts. This is in line with other studies, including the European Health Literacy survey (HLS-EU), where items reflecting more advanced, critical HL skills were experienced as the most difficult [6, 33]. In the HLS-EU, HL was as expected highest in Western European countries, but with substantial variations related to age, health, and socioeconomic status [6]. Breast cancer risk is positively associated with high socioeconomic status, and the majority of survivors in this survey had high SES. This may account for the relatively high mean HL score. The most critical HL skills for cancer survivorship may still be lacking for a substantial proportion of survivors. In line with previous findings, we observed an association between lower HL and increasing age [12, 16]. Old age involves declining sensory and cognitive functioning which may affect HL skills directly. Additionally, with age, the risk of comorbidity also increases. Multi-morbid, more fragile patients report lower HL when faced with cancer than patients with higher performance status [11], possibly because they already engage in complex self-management behaviors. Dealing with cancer in this context may exceed their overall coping capacity. Survivors of older age may therefore represent a particularly vulnerable group with regard to the effects of low HL.

FCR, the persistent fear or worry that the cancer will return or progress [34], is a frequent concern among survivors of breast cancer [35], shown to persist over time, also among survivors with favorable long-term prognosis, such as survivors of breast cancer [36]. In the present study, FCR was the only late effect that remained significantly associated with HL in the adjusted model. Our finding is in line with results presented by Halbach et al. who concluded that limited HL is an independent risk factor for higher levels of FCR [16]. Low HL may impact the risk of FCR through different mechanisms, such as reduced ability to navigate, critically assess, understand, and handle health-related information, but also through sub-optimal interaction with health care providers, for instance, through avoidance behavior. Among patients with cancer, low HL has been associated with lower perceived information provision and information satisfaction compared to higher HL [37]. Combined, low HL may result in unmet informational and supportive care needs and perhaps unnecessarily high levels of FCR, as demonstrated in previous studies [18].

In an oncological setting, neuroticism, the propensity for negative emotions when faced with negative stress, has been associated with reduced quality of life and increased risk of post-traumatic stress disorder following cancer [38]. Neuroticism may affect self-management behaviors, as reported for patients with other chronic diseases such as diabetes [39], thereby linking it to HL skills. Although research is limited, low HL has been associated with neuroticism [40]. We observed the same association, which remained stable in the adjusted analyses. We have not been able to find other studies specifically exploring HL and neuroticism among survivors of breast cancer warranting further investigations. Our finding implies that specific personality characteristics may indicate poorer HL skills. Such knowledge may be valuable in a clinical setting making it easier for clinicians to identify individuals at risk of poorer outcomes.

As symptom burden and functional limitations increase, self-perceived HL and self-management seem to decline [11]. Following this line of reasoning, individuals with low HL may be particularly vulnerable when faced with treatment-related late effects. This was not the case in the present sample. This may be due to the survivors’ relatively high HL levels, enabling them to handle these stressors, or that increasing demands in fact has stimulated HL skills over time. It is also possible that HL is more clearly linked to late effects during the first years of cancer survivorship, before effective coping strategies are in place and the emotional distress is likely to be higher. Exploring HL among patients living with advanced breast cancer will be interesting for further studies.

### Strengths and limitations

Survivors of breast cancer included in this study were recruited from an unselected, unbiased population. All long-term, early-stage survivors diagnosed with breast cancer and within working age registered in the CRN were invited to participate, granting us with a large sample of high quality.

Given the inclusion criteria for the present study, non-responders did not differ according to diagnosis (early-stage BC), gender, or time of diagnosis. Additional clinical data from the CRN concerning cancer-related variables revealed only slight differences between responders and non-responders. We cannot exclude the possibility that non-responders differ according to other variables, such as ethnic background and HL skills, as reading and understanding Norwegian were necessary in order to complete the questionnaire. However, data use restrictions prohibited us from performing complete analyses of the non-responders. Although declining response rates in health surveys in general may threaten external validity, a response rate of 49% is considered acceptable and comparable to other large-scale surveys on long-term survivors of cancer in Norway [41]. Furthermore, in comparable survey studies, with more modest response rates than reported here, evidence of response bias was not found [42].

This sample reflects the Norwegian population of breast cancer survivors, which is quite homogenous in terms of SES and ethnic background. Although the results are likely to be generalizable to a Scandinavian and Western European setting, more caution must be paid when interpreting the results within a more global context. We only included survivors of working age at diagnosis (20–65 years) given the main outcomes of interest in the SWEET study. Inclusion of the oldest age groups may have further underlined the importance of increasing age as a risk factor for poor HL and should be further explored in upcoming studies. Due to the cross-sectional design, we were unable to explore the directionality between HL and the other variables. A prospective methodology should be applied to assess these associations further. Data on HL is based on self-report and reflect present skills.

Although validated in the Norwegian population and for Norwegians with diabetes type II [20, 43], the HLS-Q12 it has not been extensively used. Furthermore, categorization of HL is empirically based, and threshold values will vary. Consequently, we chose to use HL as a continuous variable for the regression analyses. The HLS-Q47 allows for the opportunity to measure HL across three health care settings. This procedure has not been described for the HLS-Q12 as of yet. Still, the HLS-Q12 is reported to be a valid, quick, and easy-to-use tool to measure HL in a clinical setting.

## Conclusions

Within this nationwide cohort of long-term survivors of breast cancer, one in five reports HL levels within the marginal or inadequate range. Despite high SES, HL tasks specifically pertaining to advanced judgment calls were rated as difficult or very difficult by 35–41%, demonstrating that both presenting risk and understanding risk are challenging. This study demonstrates that the presence of basic HL does not automatically imply more advanced skills, which are likely to be the most crucial with regard to long-term survivorship. Sufficient individual support must be provided alongside implementing measures to improve HL at a societal and health care level. Survivors with higher age, neurotic personality traits, and reports of FCR may warrant specific attention from health care providers. It needs to be recognized that HL is likely to vary over time, with context and functional demands, and identification of individuals at risk of low HL based on objective markers alone is insufficient.

## Data Availability

All data are available at Oslo University Hospital.
